# Antipsychotics for irritability in children with Autism Spectrum Disorders

**DOI:** 10.1192/j.eurpsy.2022.1065

**Published:** 2022-09-01

**Authors:** V. Muñoz Martinez, L. Beato-Fernández, E. Segura-Escobar

**Affiliations:** 1 Hospital General Universitario de Ciudad Real, Adolescents Inpatient Unit., Ciudad Real, Spain; 2 Hospital General Universitario de Ciudad Real, Adolescents Inpatient Unit, Ciudad Real, Spain; 3 Hospital General Universitario de Ciudad Real, Chief Of Psychiatry, Ciudad Real, Spain

**Keywords:** Irritabilty, Autism Spectrum Disorder, antipsychotic, autism

## Abstract

**Introduction:**

Autism is a neurodevelopmental disorder characterized by qualitative impairments in social interaction, communication, and restricted and repetitive behaviors [1]. Despite of these symptoms, some patients present different manifestations of irritability. These can be expressed in different kinds of disruptive behaviors. Recent studies shown that, at least 20% of children with autism present irritability symptoms, which cause severe social and familiar disturbances [2].

**Objectives:**

The aim of this study was to evaluate short-term efficacy of aripiprazole in children in comparison with other antipsychotic. We include behaviors related to irritability as all kinds of aggressions, tantrums and self-injuries.

**Methods:**

90 patients were recruited. 45 of the patients received aripiprazole and 45 received other antipsychotic. The initial doses of aripiprazole was 2,5 mg/day. Doses were increase related to symptoms. The range of the doses were from 2,5 to 15 mg/day.

**Results:**

From these 45 patients 12 had a relapse (26,6%) during the next two years. From the second group, 20 (44.4%) of the patients had a relapse during the next two years. Five of the aripiprazole group (11,1%) abandon treatment. From the second group twelve patients (26.6%) also abandon treatment. Prolactin rates with aripiprazole were 28.2 ng/ml for males and 14.1 ng/ml for women.

**Conclusions:**

In general, the result of our research indicated that Aripiprazole was effective and generally safe and well tolerated in the treatment of irritability associated with ASD. One of the limitations was that we do not use scales in order to measure the changes.

**Disclosure:**

No significant relationships.

